# Prospective, non-randomized, controlled investigation of prostate (P) artery embolization (E) compared to holmium (Ho) laser enucleation of prostate for the treatment of symptomatic benign prostatic hyperplasia with prostate volume 80–250 cc: the hope trial outcome at 1 year

**DOI:** 10.1007/s00345-025-06115-0

**Published:** 2026-01-13

**Authors:** Shivank Bhatia, Ansh Bhatia, Andrew Richardson, Chloe Issa, Zachary Stauber, Kenneth Richardson, Muhammad U. Shahid, Joao G. Porto, Hemendra N. Shah

**Affiliations:** 1 Department of Interventional Radiology, University of Miami, Miller School of Medicine, 1150 NW 14 St. Suite 702, Miami, FL 33136 USA; 2 Florida Prostate Centers, 13722 Jog Rd. Suite A, Delray Beach, Miami, FL 33446 USA; 3 Desai Sethi Urology Institute, University of Miami, Miller School of Medicine, Miami, FL USA

**Keywords:** Prostate artery embolization, Holmium laser enucleation, Benign prostatic hyperplasia, Lower urinary tract symptoms

## Abstract

**Background:**

To compare outcomes of Prostate Artery Embolization (PAE) with Holmium Laser Enucleation of Prostate (HoLEP) for management of symptomatic benign prostate hyperplasia in men with prostate volume 80 to 250 cc.

**Methods:**

In this open-label prospective clinical-trial, 45 patients were enrolled in a 2:1 ratio to undergo PAE and HoLEP, respectively. The voiding parameter, sexual function, and complications were evaluated at 1, 3, 6, and 12-months. The primary endpoint was a reduction in the International Prostate Symptom Score (IPSS) at 3 months. Secondary-outcomes were change in the International Index of Erectile Function-15 (IIEF-15), International Consultation on Incontinence Questionnaire-urinary incontinence (ICIQ-UI), Quality-of-Life (QoL), IPSS, uroflow (Qmax) and Prostate Specific Antigen (PSA). Adverse-events were recorded and graded using the Clavien–Dindo Adverse-event (CD-AE) classification.

**Results:**

No significant difference in median IPSS and quality of life improvement was observed at any follow-up point. PAE had significantly better IIEF-15 and ICIQ-UI outcomes than HoLEP at 1 and 3 months. HoLEP outperformed PAE in PSA reduction, Qmax, and post-void residual (PVR) improvement at 3 and 12-months. However, PAE had shorter hospital stays and avoided catheterization. No major adverse events (Clavien–Dindo ≥ grade 3) occurred in either group.

**Conclusion:**

In men with BPH and prostate sizes 80–250gm, PAE and HoLEP produce similar IPSS and QoL improvement at medium-term follow-up. PAE had better IIEF-15 and ICIQ-UI, in addition to the advantage of avoiding catheterization and reduced hospital stay. HoLEP performed better in PSA reduction, Qmax, and PVR improvement at 3 and 12 months. No serious adverse events occurred in either treatment group. Small sample size and short-term follow-up were important limitations.

**Supplementary Information:**

The online version contains supplementary material available at 10.1007/s00345-025-06115-0.

## Introduction

Benign Prostatic Hyperplasia (BPH) affects more than 60% of men over the age of 50 and has a global financial burden exceeding $78 billion annually due to treatment costs and lost productivity in the 21 st century [1]. Approximately 8% of these men will eventually need surgical treatment for the management of LUTS or complications attributable to bladder outlet obstruction [1]. Over the past few decades, a myriad of minimally invasive surgical treatments (MIST) have emerged to manage bladder outlet obstruction (BOO) [2]. However, most treatments are limited to small or medium-sized prostates with volume < 80 cc [2]. For prostate sizes greater than 80cc, two primary options are available: simple prostatectomy and anatomic endoscopic enucleation of the prostate (AEEP). Among AEEP techniques, holmium laser-enucleation of the prostate (HoLEP) stands out as a foundational procedure. It is a proven, size-independent technique with a favorable safety profile, making it suitable for large prostates [2].

Among the minimally invasive modalities of BPH treatment, prostate artery embolization (PAE) is another size-independent modality that has gained popularity due to its safety profile and efficacy [3]. PAE has been recently included in the American Urological Association (AUA) guidelines with conditional recommendations in specific patient populations [2]. However, the guidelines also stress the need for more comparative data with established techniques and do not comment on using PAE for moderate (50–80 gm) versus large prostate sizes (>80 gm) [2].

While PAE and HoLEP have been compared individually with TURP [4, 5] and indirectly with each other [6], there have been no direct, prospective comparisons of PAE and HoLEP. The primary aim of this study is to evaluate the improvement in voiding symptoms from BPH as assessed by the International Prostate Symptom-Score (IPSS) for PAE with Embosphere^®^ Microspheres compared with HoLEP at 3-months after the procedure. The secondary aim is to evaluate functional and sexual outcomes at 3 and 12-months after the procedure.

## Materials and methods

### Study details

This was an IRB-approved (IRB-number: 20210029) prospective, open-label, non-randomized study with parallel patient-assignment assessing the functional and urological outcomes after PAE and HoLEP. The full study protocol is available on clinicaltrials.gov (NCT05155891).

### Patients

The study’s inclusion criteria were patients aged 50 and above, with a baseline IPSS ≥ 13, and possessing a prostate size ranging from 80 to 250 cc, determined via magnetic-resonance imaging (MRI) or transrectal-ultrasonography (TRUS). Patients must have demonstrated BPH symptoms resistant to medical treatment or for whom medication is unsuitable, intolerable, or refused. They were suitable candidates for HoLEP or PAE, who gave informed consent and agreed to participate in the study. Patients with indwelling urinary catheters or those performing self-catheterization were excluded. Additionally, patients with a history of prostate or bladder cancer, associated neurogenic bladder, urethral strictures, chronic prostatitis, or pelvic pain syndrome were excluded. The full inclusion/exclusion criteria can be found in supplemental Table 2.

### Procedural details

#### PAE

A single operator with over 18-years of experience performed all the PAE procedures under conscious sedation with midazolam and fentanyl. Pre-procedural antibiotic prophylaxis was administered as per the institution’s guidelines. PAE was performed via radial-access. The left radial artery was evaluated via the Barbeau-test and ultrasound. Contraindications to radial artery access included: Barbeau type-D pattern, radial artery diameter less than 2 mm, significant tortuosity and/or radial loop. Superselective-catheterization of the prostate-artery was performed in a standard manner with a 2.1-F 150-cm microcatheter (Maestro; Merit Medical Systems, South Jordan, UT) and 0.014-inch or 0.016-inch microguide wire (Fathom; Boston Scientific, Boston, MA). Non-target collateral vessels were coil-embolized (Tornado by Cook Medical, Bloomington, IN, or Concerto by Medtronic, Minneapolis, MN) when necessary. All PAEs were performed with either 100–300 μm, 300–500 μm or a combination of 100–300 μm and 300–500 μm Embosphere Microspheres (Merit Medical Systems, South Jordan, UT). Successful embolization was defined as the stasis of flow in the prostatic artery. A post-procedural cone-beam CT confirmed appropriate prostate gland embolic coverage as portrayed by contrast retention. The procedural steps have been previously published [7]. PAE patients were not catheterized post-procedure. For PAE, the time from vascular access to closure was considered the procedure duration.

Patients were discharged home on the same day (3 hours post-procedure), with a standard post-procedure medication regimen [3]. Patients continued to take their prescribed BPH medications, including 5-alpha reductase inhibitors and alpha-blockers, for at least 4-weeks after PAE. At the 1-month follow-up after PAE, patients were then instructed by the interventional-radiologist to continue or discontinue these medications depending on the patient’s response to PAE and shared decision-making.

#### HoLEP

All patients underwent “en-bloc” HoLEP as previously described [8]. The procedure was performed by a single surgeon with over 19 years of experience. Enucleation was performed with a 550-micron laser fiber using a 100–120 W holmium laser with Moses technology (Lumenis, Santa Clara, CA). Morcellation was performed with Lumenis Versacut^™^ morcellator (Lumenis, Santa Clara, CA) or Wolf-Piranha-Morcellator (Richard-Wolf, Knittlingen, Germany). For the HoLEP group, the total time spent by the patient in the operating room was considered, including anesthesia time, which was defined as operation time. The Foley catheter was kept in situ overnight. Patients were observed overnight in the hospital with continuous bladder irrigation. No traction was placed on the Foley’s catheter. Postoperatively, they were followed up at 1, 3, 6, and 12-months (± 2-weeks). BPH medication was stopped postoperatively in all patients.

### Data collection

Demographic details, Prostate-Volume (PV), IPSS, Quality-of-Life (QoL) question, International Index of Erectile-Function-15 (IIEF-15)-score, *International Consultation on Incontinence Questionnaire*-Urinary-Incontinence (ICIQ-UI), Post-Void-Residual (PVR), maximum urine-flow velocity (Qmax), Prostate-Specific-Antigen (PSA) and BPH medications usage were collected at baseline. Vascular-access, peri-procedural details, adverse effects, hospital stay, and duration of catheterization were collected after the procedure. Outcome measures were recorded per the study protocol: Change in IPSS, QoL, and IIEF-15 at 1, 3, 6, and 12-months. Change in ICIQ was measured at 1 and 3-months. Qmax and PVR were recorded at 3 and 12-months. Post-operative PSA values were measured between 3 and 12-months. Adverse events at these follow-up visits were collected and classified according to the modified Clavien-Dindo (CD) [9] adverse events classification system.

### Statistical analysis

**Fig. 1:**
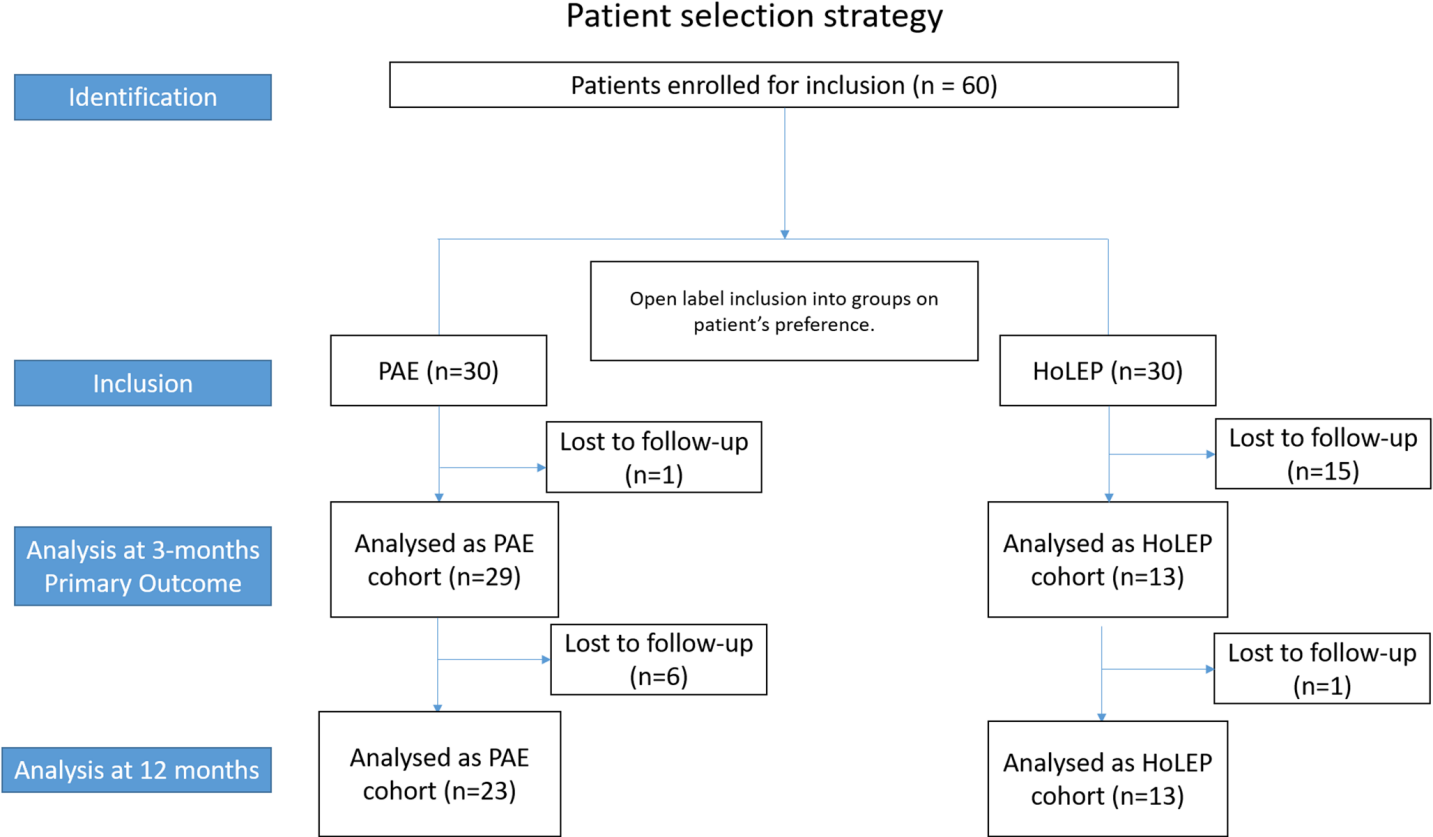
Patient selection and follow-up. Flowchart showing the patient selection strategy, loss to follow up and final analysis performed for the primary endpoint

This study initially aimed to recruit 82 patients in a 1:1 ratio for PAE and HoLEP, anticipating an 80% follow-up rate and using a minimal clinically important difference (MCID) of 3 points for IPSS. However, at the 9-month mark after the start of enrolment, a significant loss of follow-up occurred in the HoLEP group. To account for this loss, the study protocol was revised to a 2:1 ratio for PAE and HoLEP, ensuring a statistical power of over 80% based on an MCID of 4 IPSS points (Fig. 1). A minimum of 12 HoLEP and 24 PAE patients were required to adequately power the primary outcome evaluation, defined as the difference in IPSS scores at 3 months.

Continuous variables were summarized as mean ± standard-deviation or median (inter-quartile range) depending on the data distribution. Categorical-variables were summarized as counts and percentages. The Shapiro-Wilks test and visual plots were used to evaluate the normal distribution of the data. As appropriate, continuous-variables were assessed using the two-sided t-test or Wilcoxon rank-sum test. Categorical-variables were evaluated using the Chi-squared and Fisher’s exact test as appropriate. Rstudio version 4.3.3 (Rstudio Inc., Boston, MA, USA) was used for this analysis.

## Results

A total of 45 patients underwent PAE and HoLEP (30:PAE and 15:HoLEP) from July 2022 to December 2023. All patients in the PAE arm had successful bilateral embolization. At 12 months, 23 and 12 patients in the PAE and HoLEP cohorts were available for follow-up. The baseline demographics, BPH parameters, and peri-procedural variables can be seen in Table 1.

**Table 1:** Demographics of the study population, peri-procedural outcomes

	PAE	HoLEP	*P* Value
Number of patients	30	15	
Patient demographic			
Age (yr) *	68.2 ± 7.6	66.7 ± 6.3	0.42
BMI (kg/m^2^)*	28.3 ± 3.4	28.41 ± 3.6	0.83
PSA (ng/ml)*	6.5 ± 4.6	6.1 ± 4.7	0.93
Prostate Volume (ml)*	128 ± 33	120 ± 25	0.35
Procedural details			
Procedure time*	49.07 ± 17.20 min	151.3 ± 55 mins	**<0.001**
PAE procedure details			
Bilateral (N, %)	100	NA	
Unilateral (N, %)	0	NA	
Fluoroscopy time (min)*	24.0 ± 11.7	NA	
Dose area product (uGy-m^2^) †	13,161.5 (9907.2–19152.25.2.25)	NA	
Amount of embolization particles used (mL)*	15.9 ± 4.4 ml	NA	
Post-procedure parameters:			
Blood loss (cc)*	Negligible	53.3 ± 12.9 cc	
Duration of hospital stay	3 (3–3) hours	1 (1–1) days	
Weight of Resected tissue*	NA	93 ± 31 g	
Duration of catheterization (hr)*	NA	20.7 ± 6.1 h	

*Mean ± SD

^†^Median (IQR)

**Table 2:** Comparison of follow-up data between PAE and holep

Baseline data [(Mean ± SD) or Median (IQR)]	PAE arm		HoLEP arm		*p*-value
IPSS	26 (22–28)		20 (16–24)		**0.006**
QoL	5 (4–6)		5 (4–6)		0.82
PVR	106 ± 87		101 ± 58		0.81
Qmax	7.3 ± 2.5		8.5 ± 5.5		0.46
PSA	6.5 ± 4.6		6.1 ± 4.7		0.93
ICIQ Score	6 (0–11)		6 (0–9)		0.65
IIEF-15 score	35 (15–52)		45 (23–60)		0.58
**1-month follow-up [(Mean ± SD) or Median (IQR)]**	*N*=30	Median % reduction from baseline	*N*=15	Median % reduction from baseline	
IPSS	−19 (−22 to −15)	−73%	−16 (−19 to −10)	−80%	0.1
QoL	−4 (−5 to −3)	−80%	−3 (−4 to −2)	−60%	0.11
IIEF-5 score	0 (−2 to 13)	0%	−45 (−54 to −26)	−100%	**0.001**
ICIQ score	−3 (−8 to 0)	− 50%	2 (−6 to 7)	+ 33%	**0.037**
**3-month follow-up [(Mean ± SD) or Median (IQR)]**	*N*=29	Median % reduction from baseline	*N*=13	Median % reduction from baseline	
IPSS	−20 (−21 to −17)	−77%	−17 (−21 to −13)	−85%	0.24
QoL	−4 (−5 to −3)	−80%	−3 (−5 to −1)	−60%	0.23
PVR	81 ± 101	23.5%	21 ± 16	79.2%	**0.01**
Qmax (change from baseline)	+ 6.7 ± 7.0	+91.8%	+ 12.8 ± 6.9	150.6%	**0.02**
PSA	2.69 ± 2.25	−58.4%	0.41 ± 0.33	− 93.4%	**<0.001**
IIEF-5 score	5 (0 to 16)	+14.3%	−7 (−26 to 9)	−15.5%	**0.047**
ICIQ score	− 2 (−8 to 0)	−33%	0 (−2 to 3)	NA	**0.013**
**6-month follow-up [(Mean ± SD) or Median (IQR)]**	*N*= 25		*N* = 13		
IPSS	−22 (−24 to −16)	− 84.6%	−17 (−21 to −14)	− 85%	0.093
QoL	− 4 (−5 to −3)	− 80%	−3 (−3 to −2)	− 60%	0.063
IIEF-5 score	6 (1–22)	+ 17%	1 (−4 to 4)	+0.02%	0.077
**12-month follow-up [(Mean ± SD) or Median (IQR)]**	*N* = 23	Median % reduction from baseline	*N* = 12	Median % reduction from baseline	
IPSS	−17 (− 21 to – 15)	− 65.4%	− 16 (− 21 to − 12)	− 80%	0.33
QoL	− 3 (−4 to −2)	−60%	− 3 (−4 to −3)	−60%	0.38
IIEF-5 score	1 (−2 to 14)	+2.9%	0 (−2 to 6)	0%	NA*
PSA	4.0 ± 2.7	+ 38.5%	0.5 ± 0.4 †	+91.8%	**<0.001**
PVR	43.5 ± 38 (*N*=14)	−58.5%	23 ± 36.7 (*N*=13)	−77.2%	0.19
Qmax	+3.2 ± 4.7	+43.8%	+17.1 ± 9.2	+201%	<**0.001**
**Medical Management at baseline (n)**					
5alpha reductase	9		2		0.563
Alpha-blockers	22		12		
Anti-muscarinic agents	0		1		
PDE-5 inhibitors	8		2		
**Medical Management at 3 months (n)**					
5alpha reductase	0		0		<0.001
Alpha-agonists	13		0		
Anti-muscarinic agents	0		0		
PDE-5 inhibitors	7		0		

*The sample size is too small to be adequately powered for a meaningful comparison. Therefore, no p-value has been reported

^†^*N* = 7, with 2 patients only having 6 month PSA data available

**Fig. 2:**
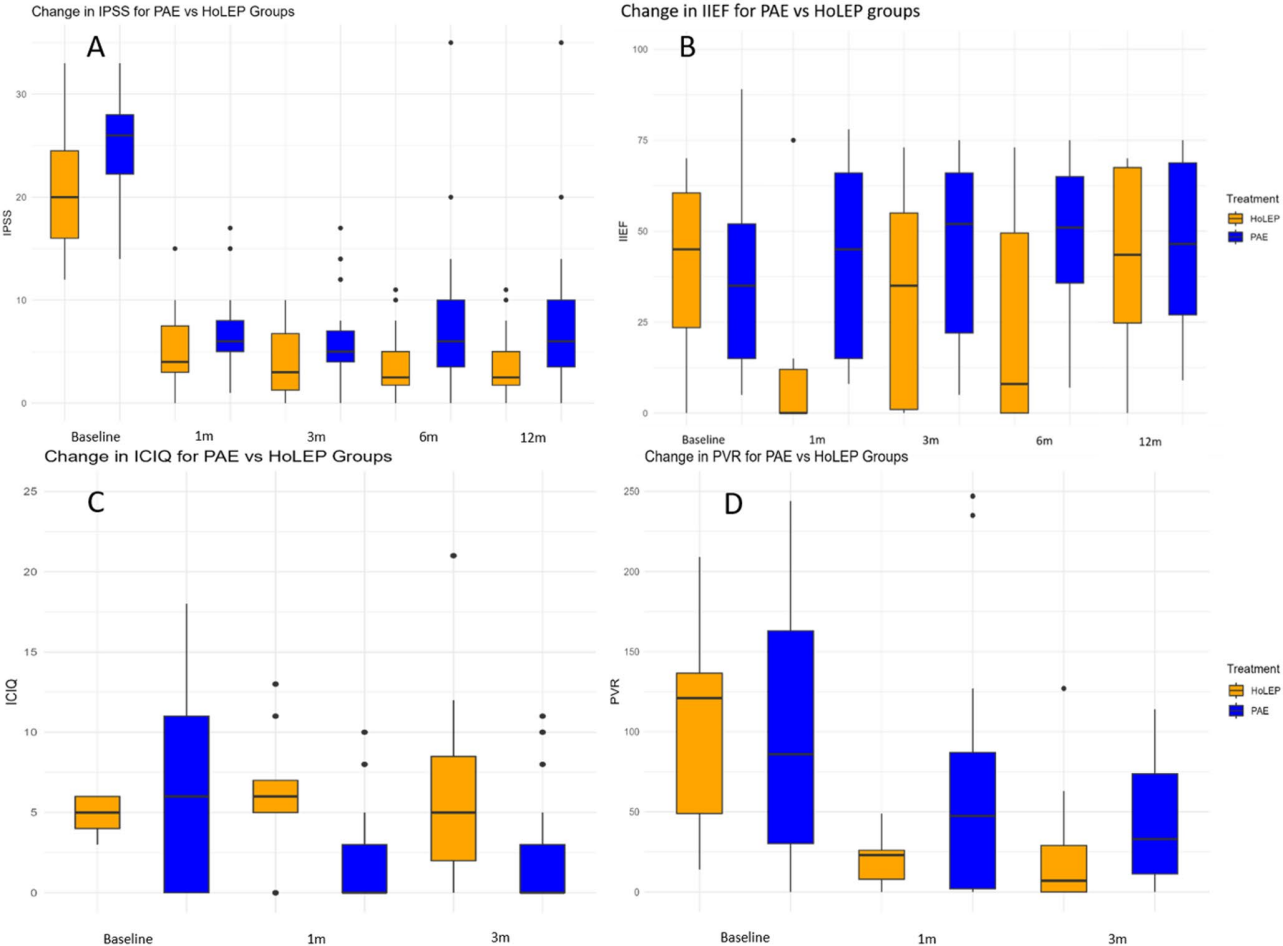
Functional outcomes after PAE and HoLEP. Clockwise from the top left; **A** International Prostate Symptom Score (IPSS) comparison between Prostate Artery Embolization (PAE) and Holmium Laser Enucleation of Prostate (HoLEP) up to 12 months. **B** International Index of Erectile Function-15 (IIEF-15) comparison between PAE and HoLEP up to 12-months. **C** International Consultation on Incontinence Questionnaire-Urinary Incontinence (ICIQ-UI) comparison between PAE and HoLEP. **D** Post-Void Residual (PVR) outcomes comparison between PAE and HoLEP

For the primary outcome, the median (IQR) IPSS reduction was 20 (17 to 21) and 17 (13 to 21) for the PAE and HOLEP groups, respectively (*p* = 0.24). Detailed analysis of other outcomes can be seen in Table 2. This study found that PAE and HoLEP had similar reductions in IPSS and QoL at 1, 3, 6, and 12-months (Table 2). The IIEF-15 significantly worsened with HoLEP compared to PAE at 1 and 3-months. The ICIQ-UI score significantly worsened with HoLEP compared to PAE at 1 and 3-months. HoLEP had significantly better PVR, Qmax, and PSA improvement than PAE at 3-months (Table 2). The median prostate volume was 95 ± 32.33 ml at 3-months after PAE compared to 127.8 ± 32.7 ml at baseline (34.5% decrease). Post-operative imaging for prostate size measurement was not available for the patients in the HoLEP group. The urological outcomes can be visualized in Fig. 2. A 12-month follow-up is also presented in Table 2.

Fifteen (50%) patients in the PAE group experience post-embolization syndrome. This was not considered an adverse event. Similarly, mild haematuria after HoLEP was considered a normal part of recovery, not an adverse event. No patient in either group experienced a serious adverse event (CD-AE Grade ≥ 3). No patient in the PAE arm had non-target embolization. Similarly, no patient in the HoLEP arm needed a blood transfusion or re-catheterization, had a UTI, or developed urethral stricture or bladder neck stenosis during the follow-up period. Histopathological examination of resected prostate tissue after HoLEP showed BPH in all 15 patients. BPH medication was higher in the PAE group at 3 months (Table 2). No patient in either group needed surgical retreatment at 1-year follow-up.

## Discussion

In this prospective, comparative study we noted that both PAE and HoLEP were equally effective in reducing the subjective measures of voiding symptoms (IPSS and QOL) at 1, 3, 6, and 12 months. Both procedures had minimal adverse events, while PAE was better at preserving continence at 3 and 6 months. HoLEP performed better than PAE in Qmax improvement and PSA reduction at 3 months. PVR improvement was better with HoLEP at 3 months but was similar in both groups by 12 months.

IPSS improvement is the primary goal in the management of LUTS. The present study reported a median improvement of 20 (77%) and 17 points (85%) from baseline at 3-months after PAE and HoLEP, respectively, which was the primary endpoint. We noted that IPSS reduction for PAE remained comparable to HoLEP at 6 and 12-months. A Randomized Controlled Trial (RCT) with 100 patients comparing PAE and TURP reported that the IPSS improvement from baseline was 9.2 (48%) and 9.3-points (48%) after PAE and 10.8 (61%) and 11.8 points (67%) after TURP at 3 and 12-months [4, 10], respectively. Another RCT with 45-patients reported a 21-point (81%) IPSS improvement after PAE compared to 18.2 points (70%) with TURP at 1 year [11]. In our study, the change in IPSS from baseline is 17 (65%) points after PAE and 16points (80%) after HOLEP at 12 months, comparable to the improvement reported by previous studies [11, 12].

An RCT with 164-patients in a 1:1 ratio reported that the IPSS improved by approximately 18 points (80%) from baseline after both HoLEP and TURP [13] at 1 month. An RCT with 60 patients in a 1:1 ratio for HoLEP and TURP reported that the IPSS improved by 21 points (81%) after HoLEP and 20 points (84%) after TURP at 3 months [14]. A network meta-analysis (NMA) comparing PAE and HoLEP with TURP as a comparator showed that both treatments had no statistically significant difference in IPSS improvement at 3 and 12-months [15].

This study showed that the median QoL improvement was similar at 3–4 points for both treatments at 1, 3, 6, and 12-months. QoL improvement from baseline after PAE has been reported to be 3–4 points (60–80%) at 3 and 12-months in similar studies [10]. HoLEP has also been shown to improve QoL by 3–4 points (60–80%) from baseline at 3 and 12-months [13]. In line with our findings, an NMA comparing PAE and HoLEP showed that both treatments had no statistically significant difference in QoL improvement at 3 and 12-months [15].

In the present study, PVR-improvement was statistically significantly better in the HoLEP arm at 3-months but was not clinically significant. PVR Improvement was similar at 12-months between PAE and HoLEP. Previous studies have found that PVR-improvement with PAE is approximately 40–50 ml from baseline at 3 and 12-months [16]. HoLEP has been shown to improve PVR by up to 100 ml from baseline and is comparable to TURP for PVR improvement up to 12-months [17]. PVR improvement is similar in some cases in PAE, TURP, and HoLEP at 3 and 12-months [15]. Similarly, Qmax improvement was significantly better in the HoLEP cohort (150% from baseline) compared to the PAE (92% from baseline) cohort at 3 months in this study. Previous studies comparing PAE with TURP have shown that Qmax after PAE improved by 73% while Qmax after TURP improved by 210% from baseline at 3 months [4]. Previous studies comparing HoLEP with TURP have shown that Qmax improvement was 218% after TURP and 247% after HoLEP at 1 month, a clinically insignificant difference [13]. A network meta-analysis found that HoLEP outperformed PAE by a median Qmax improvement of 8.4 ml/s at 3 months [15]. We believe that contrary to HoLEP, which results in immediate tissue removal, PAE causes a gradual reduction in prostate volume, thus explaining delayed improvement in PVR and Qmax after PAE compared to HoLEP [15]. In line with this, a cohort study showed that PSA (a surrogate marker of prostate volume) steadily reduced until 24–36 months after PAE [3].

Prostate volume reduction after PAE was approximately 30 ml (34.5%) at 3-months compared to baseline in this study. A meta-analysis of 19 studies showed that the total reduction in prostate volume was similar to our study of 30 ml at 3 months after PAE. Our study did not measure prostate volume after HoLEP. However, other studies have shown that the mean PV post-HoLEP is below 30 ml at 6 months [14]. The present study found that the PSA drop was significantly more with HoLEP (92.6% decrease from baseline) compared to PAE (58.4% decrease from baseline) at 3 months. Considering PSA as a surrogate marker of residual prostate volume, this PSA reduction reflects the likely decrease in prostate size. PSA reduction in PAE trials has been reported at 20–40%, with the nadir at 6 months [18] and 85% after HoLEP at 3–6 months [8].

We found that the ICIQ-UI improved with patients who underwent PAE and worsened with those who underwent HoLEP at 1 and 3 months, indicating that PAE is better suited to preserve continence in the short post-operative period. One study that evaluated the ICIQ-UI as a secondary outcome after PAE reported an improvement of approximately 4 points at one month from baseline [19]. Transient urinary incontinence is well known after enucleation procedures, with an incidence rate of 4% at 1–3 months [20]. Newer techniques that preserve urinary continence have reported improved continence rates post-HoLEP.

The present study reported that the IIEF-15 score remained stable after PAE but significantly worsened after HoLEP at 1 and 3 months. This is partly because patients are asked to hold a sexual activity for the first month after HoLEP at our institution. The IIEF-15 score was not significantly different at 6 months (Table 2). A recent RCT comparing PAE to medication therapy showed that while the IIEF-15 score after PAE remained stable, it worsened by approximately 3 points after 9-months of medication therapy [21]. Other studies have reported that the IIEF-15 score worsens after HoLEP, usually due to HoLEP’s negative impact on IIEF-15 domains related to ejaculatory function [22]. A systematic review concluded that while evidence regarding changes in IIEF-15 scores following enucleation procedures is inconsistent, the ejaculatory function is likely negatively impacted by enucleation procedures [23]. Newer templates of enucleation to preserve ejaculation after HoLEP are being developed. Regarding erectile function, a review found that PAE is not associated with worsening erectile function in most patients and is associated with improved erectile function in up to 32% of patients [24]. Current HoLEP techniques are unlikely to impact erectile function in most patients [25].

At 1-year follow-up, no patients in either group have undergone surgical retreatment. A study assessing long-term PAE outcomes reported that the surgical retreatment rate after PAE is approximately 3–4% and 16% [3] at 1 and 5-years, respectively. The surgical retreatment rate after HoLEP has been reported to be 2–3% at 4–5% after 5-years [26]. In our study, BPH medication use was higher in the PAE group at 3-months (43%) compared to the HoLEP group (0%). Medical retreatment rates after PAE have been reported to be between 30 and 40% after PAE at 1 year in large cohort studies, which are likely to provide a reliable estimate of medication use [3]. In the recent PARTEM trial, 7% of participants in the PAE arm required BPH medication at 9 months after randomization [21]. In our study, a subset of PAE patients continued medical therapy at follow-up, which likely confound the IIEF-15 and IPSS improvement in this group. This reflects real-world clinical practice, where PAE patients are often maintained on adjunctive medical therapy during the recovery phase, while HoLEP typically allows complete discontinuation.

This study found no serious adverse events (CD-AE criteria ≥ grade 3) in either group. Both groups experienced self-limited pain, burning, urgency, pelvic pain, and transient hematuria in the immediate postoperative period, which were self-limited. Historically, PAE has had considerably lower adverse event rates than surgical treatments such as TURP [4] due to its minimally invasive nature. Access site-related complications after PAE are encountered in less than 3% of patients and may be more serious with radial access [27]. A large retrospective cohort study reported common adverse events after PAE, including UTI (1.3%), hematuria (5%), urinary retention (0.3%), and non-target embolization (4%) [16]. Adverse events after HoLEP commonly include UTI (10–13%), hematuria (1–5%), urinary retention (7–11%), and blood transfusion (0.5–1.5%) [28, 29]. In the present study, none of the patients had these complications. Quantitative analysis has shown that HoLEP and PAE are associated with fewer adverse effects than TURP [15]. Network meta-analysis has shown that PAE has lower adverse event rates than HoLEP [15].

### Limitations and future research

This study has several limitations. First, it was conducted at a single center, and both PAE and HoLEP were performed by highly experienced operators, which may limit generalizability. Second, the non-randomized design introduces the potential for selection bias. Although randomization would theoretically provide a more rigorous comparison, prior attempts to randomize PAE versus endoscopic surgery in the United States have faced major recruitment challenges, making real-world randomization difficult to achieve. Third, we experienced notable loss to follow-up, which required protocol adjustment and may have affected the completeness of outcome assessment. Finally, a subset of patients in the PAE group continued medical therapy at follow-up, which may have contributed to improvements in IPSS and IIEF-15, confounding the interpretation of treatment effect in this cohort. Despite these limitations, this is the first prospective study directly comparing HoLEP and PAE for prostates > 80 cc in a real-world clinical setting, providing practical insight into treatment selection and outcomes. Future studies should prioritize multi-center designs with strategies to minimize attrition and account for continued medication use to better define comparative outcomes.

## Conclusion

When comparing PAE and HoLEP for BPH with prostate volume > 80 cc, both procedures produced similar IPSS and QoL improvements without any serious adverse events at 1 year follow-up. HoLEP resulted in superior objective functional outcomes, whereas PAE demonstrated better IIEF-15 and ICIQ-UI scores, shorter hospitalization, and avoided catheterization. PAE remains a reasonable alternative for patients with large prostates, particularly those prioritizing a minimally invasive approach, though with higher likelihood of ongoing medical therapy or retreatment.

## Electronic supplementary material

Below is the link to the electronic supplementary material.

Supplementary Material 1

## Data Availability

The patient level data is available on reasonable request to the authors.

## References

[1] Saigal CS, Movassaghi M, Pace J et al (2007) Economic evaluation of treatment strategies for benign prostatic hyperplasia–is medical therapy more costly in the long run? J Urol 177:1463–1467 discussion 1467 17382755 10.1016/j.juro.2006.11.083

[2] Sandhu JS, Bixler BR, Dahm P et al (2024) Management of lower urinary tract symptoms attributed to benign prostatic hyperplasia (BPH): AUA guideline amendment 2023. J Urol 211:11–19 37706750 10.1097/JU.0000000000003698

[3] Bhatia S, Bhatia A, Richardson AJ et al Prostatic artery embolization: Mid- to Long-Term outcomes in 1,075 patients. J Vasc Interv Radiol 2024: S1051-0443(24)00698–5. 10.1016/j.jvir.2024.11.002 39532156

[4] Abt D, Hechelhammer L, Müllhaupt G et al (2018) Comparison of prostatic artery embolisation (PAE) versus transurethral resection of the prostate (TURP) for benign prostatic hyperplasia: randomised, open label, non-inferiority trial. BMJ 361:k2338 29921613 10.1136/bmj.k2338 PMC6006990

[5] Kuntz RM, Lehrich K, Ahyai S (2004) Transurethral holmium laser enucleation of the prostate compared with transvesical open prostatectomy: 18-month follow-up of a randomized trial. J Endourol 18:189–191 15072629 10.1089/089277904322959851

[6] Bhatia A, Porto JG, Maini A et al (2023) One-year outcomes after prostate artery embolization versus laser enucleation: A network meta-analysis. BJUI Compass n/a. Available at: https://onlinelibrary.wiley.com/doi/abs/10.1002/bco2.302, accessed November 14 10.1002/bco2.302 PMC10869668 38371212

[7] Bhatia A, Maini A, Bhatia S (2022) Prostatic artery embolization: technical pearls. Semin Intervent Radiol 39:555–561 36561798 10.1055/s-0042-1759690 PMC9767765

[8] Martos M, Katz JE, Parmar M et al (2021) Impact of perioperative factors on nadir serum prostate-specific antigen levels after holmium laser enucleation of prostate. BJUI Compass 2:202–210 35475131 10.1002/bco2.68 PMC8988639

[9] Dindo D, Demartines N, Clavien P-A (2004) Classification of surgical complications: a new proposal with evaluation in a cohort of 6336 patients and results of a survey. Ann Surg 240:205 15273542 10.1097/01.sla.0000133083.54934.ae PMC1360123

[10] Abt D, Müllhaupt G, Hechelhammer L et al (2021) Prostatic artery embolisation versus transurethral resection of the prostate for benign prostatic hyperplasia: 2-yr outcomes of a randomised, open-label, single-centre trial. Eur Urol 80:34–42 33612376 10.1016/j.eururo.2021.02.008

[11] Insausti I, Ocáriz ASde, Galbete A et al (2020) Randomized comparison of prostatic artery embolization versus transurethral resection of the prostate for treatment of benign prostatic hyperplasia. J Vasc Interv Radiol 31:882–890 32249193 10.1016/j.jvir.2019.12.810

[12] Carnevale FC, Iscaife A, Yoshinaga EM et al (2016) Transurethral resection of the prostate (TURP) versus original and perfected prostate artery embolization (PAE) due to benign prostatic hyperplasia (BPH): preliminary results of a single center, prospective, urodynamic-controlled analysis. Cardiovasc Intervent Radiol 39:44–52 26506952 10.1007/s00270-015-1202-4

[13] Sun N, Fu Y, Tian T et al (2014) Holmium laser enucleation of the prostate versus transurethral resection of the prostate: a randomized clinical trial. Int Urol Nephrol 46:1277–1282 24492988 10.1007/s11255-014-0646-9

[14] Wilson LC, Gilling PJ, Williams A et al (2006) A randomised trial comparing holmium laser enucleation versus transurethral resection in the treatment of prostates larger than 40Grams: results at 2 years. Eur Urol 50:569–573 16704894 10.1016/j.eururo.2006.04.002

[15] Bhatia A, Porto JG, Maini A et al (2024) One-year outcomes after prostate artery embolization versus laser enucleation: a network meta-analysis. BJUI Compass 5:189–206 38371212 10.1002/bco2.302 PMC10869668

[16] Bilhim T, Costa NV, Torres D et al (2022) Long-term outcome of prostatic artery embolization for patients with benign prostatic hyperplasia: single-centre retrospective study in 1072 patients over a 10-year period. Cardiovasc Intervent Radiol 45:1324–1336 35778579 10.1007/s00270-022-03199-8

[17] Cornu J-N, Ahyai S, Bachmann A et al (2015) A systematic review and meta-analysis of functional outcomes and complications following transurethral procedures for lower urinary tract symptoms resulting from benign prostatic obstruction: an update. Eur Urol 67:1066–1096 24972732 10.1016/j.eururo.2014.06.017

[18] Mouli S, Salem R, McClure TD (2025) Prostate Artery Embolization for Benign Prostatic Hyperplasia. J Urol 2024. Available at: https://www.auajournals.org/doi/10.1097/JU.0000000000003976, accessed February 15 10.1097/JU.0000000000003976 PMC12721628 38703386

[19] Schmidt VF, Schirren M, Heimer MM et al (2022) Semi-Automatic MRI feature assessment in Small- and Medium-Volume benign prostatic hyperplasia after prostatic artery embolization. Diagnostics (Basel) 12:585 35328138 10.3390/diagnostics12030585 PMC8946889

[20] Hout M, Gurayah A, Arbelaez MCS et al (2022) Incidence and risk factors for postoperative urinary incontinence after various prostate enucleation procedures: systemic review and meta-analysis of pubmed literature from 2000 to 2021. World J Urol 40:2731–2745 36194286 10.1007/s00345-022-04174-1

[21] Sapoval M, Thiounn N, Descazeaud A et al (2023) Prostatic artery embolisation versus medical treatment in patients with benign prostatic hyperplasia (PARTEM): a randomised, multicentre, open-label, phase 3, superiority trial. The Lancet Regional Health – Europe : ; 31. Available at: https://www.thelancet.com/journals/lanepe/article/PIIS2666-7762(23)00091-1/fulltext, accessed January 21, 2024 10.1016/j.lanepe.2023.100672 PMC10320610 37415648

[22] Pushkar P, Taneja R, Agarwal A (2019) A prospective study to compare changes in male sexual function following holmium laser enucleation of prostate versus transurethral resection of prostate. Urol Ann 11:27–32 30787567 10.4103/UA.UA_44_18 PMC6362791

[23] Cheng BK-C, Li TC-F, Yu CH-T (2020) Sexual outcomes of endoscopic enucleation of prostate. Andrologia 52:e13724 32557813 10.1111/and.13724

[24] Wong T, Tembelis M, Acharya V et al (2020) Prostatic Artery Embolization and Sexual Function: Literature Review and Comparison to Other Urologic Interventions. Techniques in Vascular & Interventional Radiology : ; 23. Available at: https://www.techvir.com/article/S1089-2516(20)30042-1/fulltext, accessed March 14, 2022 10.1016/j.tvir.2020.100693 33308525

[25] Roper C, Slade A, Caras R et al (2024) Ejaculatory and erectile function outcomes following holmium laser enucleation of the prostate. Prostate 84:791–796 38558096 10.1002/pros.24697

[26] Elzayat EA, Elhilali MM (2007) Holmium laser enucleation of the prostate (HoLEP): long-term results, reoperation rate, and possible impact of the learning curve. Eur Urol 52:1465–1472 17498867 10.1016/j.eururo.2007.04.074

[27] Richardson AJ, Kumar J, Richardson K et al (2023) Safety of prostate artery embolization via transradial access versus transfemoral access. J Vasc Int Radiol; 0. Available at: https://www.jvir.org/article/S1051-0443(23)00879-5/fulltext, accessed January 8, 2024 10.1016/j.jvir.2023.09.036 38103863

[28] Hines L, Doersch KM, Ninomiya M et al (2023) Redefining clinically significant hematuria after holmium enucleation of the prostate. J Endourol 37:1216–1220 37725558 10.1089/end.2023.0317

[29] Elsaqa M, Dowd K, El Mekresh A et al (2023) Predictors of postoperative urinary tract infection following holmium laser enucleation of the prostate. Can Urol Assoc J 17:E364–E368 37549346 10.5489/cuaj.8269 PMC10657227

