# Supplementary material for: Prospective, non-randomized, controlled investigation of prostate (P) artery embolization (E) compared to holmium (Ho) laser enucleation of prostate for the treatment of symptomatic benign prostatic hyperplasia with prostate volume 80–250 cc: the hope trial outcome at 1 year
Source: World J Urol. 2026 Jan 13;44(1):87. doi: 10.1007/s00345-025-06115-0 (PMC12799645; doi:10.1007/s00345-025-06115-0)
Supplement: Supplementary file 1 — Supplementary Material 1 [file 345_2025_6115_MOESM1_ESM.docx]

| **Participants** | **Intervention** | **Comparator/ Outcome importance.** | **Outcomes** | **Description of Outcome** |
| --- | --- | --- | --- | --- |
| Adult males with moderate to severe LUTS (IPSS>8) due to BPH [See Supplementary table 2 for complete inclusion exclusion criteria] | PAE | HoLEP   Secondary outcome: IPSS at 1, 6, 12 months | Primary outcome International Prostate Symptom Score (IPSS) at 3 months. | Assesses the quality of life or bother score based on the patient’s perception of the problem. |
|  |  | Secondary outcome | Quality of Life Score (QoL) at 1, 3, 6, 12 months | A subset of the IPSS - determining the impact of BPH symptoms on the patient’s quality of life. |
|  |  | Secondary outcome | International Index of Erectile Function (IIEF - 15) at 1, 3, 6, 12 months | Validated questionnaire assessing the total male sexual health spectrum. |
|  |  | Secondary outcome | Post-void residual volume (PVR) at 1, 3, and 12 months | Volume of urine left in the bladder after a normal micturition. |
|  |  | Secondary outcome | Qmax at 3 and 12 months. | Peak urine flow velocity. |
|  |  | Secondary outcome | International Consultation on Incontinence Questionnaire – Urinary Incontinence (ICIQ-UI) at 1 and 3 months. | Four-item questionnaire composed of three scored items and an unscored Self-diagnostic item that assesses the prevalence, frequency, and perceived cause of urinary incontinence and its impact on everyday life. A higher score indicates worse incontinence |
|  |  | Secondary outcome | CD-AE adverse event classification system. | Adverse event classification system ^9^. Serious adverse events (requiring hospitalization) are CD≥3 |
|  |  | Secondary outcome | Duration of hospitalization after the procedure | Duration from procedure to discharge |
|  |  | Secondary outcome | Duration of urinary catheterization after the procedure | Duration of indwelling urinary catheter post-procedure. |

Supplementary table 1: PICOS – Participant, Intervention, Comparator, Outcomes

**Supplementary Table 2**

| Inclusion Criteria | Exclusion Criteria |
| --- | --- |
| 1. Patient is age 50 or older. 2. Patient has signed informed consent and agrees to attend all follow-up study visits. 3. Patient has had LUTS secondary to BPH or any complications secondary to BPH and qualifying for active intervention. 4. Patient has a baseline IPSS Score > 13 at baseline. 5. Patient has a prostate size of at least 80 grams and not more than 250 grams, measured by magnetic resonance imaging (MRI) or transrectal ultrasonography (TRUS). 6. Patient has BPH symptoms refractory to medical treatment or for whom medication is contraindicated, not tolerated, or refused. 7. Patient must be a candidate for HoLEP or PAE. | 1. Subject has untreated active infection (e.g., active urinary tract infection or prostatitis) 2. Patient has a diagnosis or received treatment for chronic prostatitis or chronic pelvic pain syndrome (e.g., nonbacterial chronic prostatitis). 3. Patients with indwelling urinary catheters or those performing self-catheterization. 4. Biopsy proven prostate or bladder cancer. Patient with elevated PSA will be counselled by urologist and a shared decision will be made with the patient after discussion about pros and cons of prostate biopsy. 5. Patients with neurogenic bladder disorder. 6. Urethral stricture, bladder neck contracture, sphincter abnormalities, urinary obstruction due to causes other than BPH, or other potentially confounding bladder or urethral disease or condition. 7. Patients with prior history transurethral resection of the prostate (TURP), Green Light laser treatment, or other prostate surgical treatments within past year (12months). 8. Any known condition that limits catheter-based intervention or is a contraindication for embolization, such as intolerance to a vessel occlusion procedure or severe atherosclerosis. 9. Cardiac condition including congestive heart failure or arrhythmia, uncontrolled diabetes mellitus, significant respiratory disease or known immunosuppression which required hospitalization within the previous 6 months. 10. Acute myocardial infarction, open heart surgery, or cardiac arrest within 180 days prior to the date of informed consent. 11. Patient is interested in future fertility and wish to preserve ejaculation will be excluded from HoLEP arm 12. History of coagulation cascade disorders (which are not normalized by medical treatment before the procedure) or disorders that affect platelet count or function (e.g., von Willebrand disease) that would put the patient at risk for intraoperative or postoperative bleeding. 13. History of major allergic reaction to iodinated contrast agents will be excluded from PAE arm. 14. History of hypersensitivity to gelatin products will be excluded from PAE arm. 15. Subject has a life expectancy of less than 2 yrs. 16. Post void residual more than 500 ml at baseline. 17. Participation in any other BPH trials during the time of study. |

**Supplementary Table 2: Eligibility criteria showing the inclusion and exclusion metrics used.**
